# Laboratory Methods in Relation to Tuberculosis

**Published:** 1913-03-15

**Authors:** 


### THE CLINICAL LABORATORY.

### IV. Laboratory Methods in Relation to Tuberculosis.

One of the most important functions of the clinical laboratory is to assist in the diagnosis of tuberculosis at an early stage. This is notoriously difficult. The physician meets with a combination of symptoms with physical signs which are merely suggestive. The symptoms may be but the aftermath of an attack of influenza or of a prolonged period of overwork, whilst the signs are often merely an exaggeration of what may be quite physiological. Authorities differ widely as to the exact significance of many slight departures from the normal physical signs in the chest. Yet all differences vanish once bacilli can be demonstrated in the sputum or elsewhere.

The problem of the clinical pathologist is, therefore, to find and use the method which will demonstrate the organism, wherever it may be, at the earliest possible moment. There are other laboratory tests which tell for or against a diagnosis of tuberculosis. All these pathological methods of diagnosis may be discussed under four headings:—

1. The demonstration of the bacilli in excretions or in parts of tissues removed for purposes of diagnosis.

2. Cytological and chemical methods of examination in the case of excretions and of puncture fluids removed from various cavities.

3. Serological examinations of the blood by means of the various "immunity reactions," such as agglutination, precipitations, complement fixation, and finally the opsonic index.

4. Histological examination of tissues where the bacilli are too scanty for demonstration.

### How to Demonstrate the Bacilli.

The recognition of the bacillus depends ultimately upon either its staining and morphological properties or the results of animal inoculation. With regard to the morphological characters, much depends on the method of staining. The old Ziehl-Neelsen method must still be acknowledged to be the best. When care is taken to overstain the preparation by boiling the stain on the slide for three or four minutes (taking care to add fresh stain to prevent drying), this method is by far the most sensitive. The possible sources of error are three in number. The first is that particles of fuchsin, scratches in the glass, and other artefacts may be mistaken for bacilli especially by the beginner. This can only be avoided by experience. The second possibility is that where microscopical slides are used a second time some bacilli may have been fixed on the glass and escaped the cleaning process. The only remedy for this is to insist that every slide which has contained bacilli shall be either stored in a proper cabinet or broken immediately after examination. The third possibility is that some other forms of "acid-fast" bacilli may be mistaken for the tubercle bacillus. The smegma bacillus, which inhabits the external genitals of both sexes, is one of the chief sources of error. Staining methods have been elaborated which are claimed to differentiate between the tubercle bacillus and others of this group, but they are unsatisfactory. There is only one means by which error can be excluded, and that is to make certain that the specimen has not been contaminated. This is possible in the case of the urine.

### Two Methods of Staining.

There are other methods of staining which have received attention of late years. Two are worthy of mention—namely, Much's method and Spengler's pikrin method. The former is essentially an intensification of Gram's staining. Its advocates claim that it will render visible forms of the tubercle bacillus which are not revealed by Ziehl's method. The general trend of pathological opinion is opposed to Much's views, and experienced workers declare it inferior to the older one. One possible source of error in Much's technique is the fact that it stains organisms other than the tubercle bacillus; if these organisms are mistaken for tubercle bacilli we have at once an explanation of its vaunted superiority. Spengler's pikrin method is a modification of Ziehl-Neelsen's technique, whereby the decolorisation is performed by picric and nitric acids. It has not yet received any strong confirmation, but is claimed to reveal bacilli where other methods have failed.

Animal inoculation is by far the most sensitive test for the presence of tubercle bacilli, for quantitative experiments have shown that six bacilli (or less) form a lethal dose for the guinea-pig. It seems to the writer that the method should receive more attention as a diagnostic measure. The fact that it is so rarely used for clinical diagnosis depends on certain difficulties which can easily be removed. The expense is often allowed to stand in the way; but infinitely greater expense is incurred by a mistake of diagnosis. The fact that for performance of the test a vivisection licence and an equipment for animal experiments are necessary will always prevent it becoming part of the routine work of most hospital clinical laboratories. The difficulties of technique have been largely overcome of recent years. The association of septicæmia-producing organisms with the tubercle bacillus in mixed infections formerly limited the utility of the method, as the animal died in a few days from sepsis; but these organisms can now be removed by the antiformin treatment. The length of time necessary for the development of tuberculosis in the animal has always been an obstacle, but it is now stated that the examination of the glands which drain the site of injection will give a positive result within fourteen days. The inoculation of the guinea-pig is the one method which demonstrates beyond criticism the presence of living tubercle bacilli. It has been in routine use for many years for the diagnosis of tuberculosis of the udder in cattle, and has been clearly proved to be superior to any microscopical method for this purpose.

The presence of lymphocytes in large numbers in pleural effusions is strong evidence of tuberculosis; but animal inoculation is a far better test. In the cerebro-spinal fluid a lymphocytosis is strong confirmatory evidence of tuberculosis when there are other signs of meningitis and when syphilis can be excluded. A lymphocytic sputum has even been claimed as evidence of early tuberculosis of the lungs, but this lacks confirmation. Various chemical tests have, at different times, been put forward as a means of identification of the tuberculous nature of sputum and exudates, but up to the present none have been proved to have any general utility.

In the case of tuberculosis there are many special difficulties in the interpretation of any immunity reactions. In the first place, we do not know what special part the serum plays in the reaction of the host to the invasion of the bacillus. There is good clinical evidence to suggest that part of the reaction is largely mechanical, and due to fibrosis in the tissues surrounding the focus of disease. The fact that by suitable treatment a serum can be obtained (from experimental animals), which has a neutralising action on the toxic properties of tuberculins is evidence that the serum participates to some extent in the conflict between organism and host. The existence of these two factors suggests an explanation of the conflicting results obtained by different investigators of the "immunity reactions." Agglutination experiments were so successful in the typhoid diagnosis that much attention was paid to the same phenomena in tuberculosis. Positive results have been obtained, but have not been found useful for diagnosis and, further, it has been definitely shown that the quantitative variations in agglutinin-content of the serum has no definite relationship to clinical phenomena. The same applies to the investigation of precipitins. The method of "complement deviation" has also shown the presence of an immunity reaction, but only in advanced cases.

Where bacilli cannot be demonstrated it is often very difficult to give a positive opinion from the histological appearances. The very chronic forms of the disease, where almost the sole lesion consists of fibrosis, are very difficult to distinguish from other lesions, whilst, on the other hand, it is stated that the tubercle bacillus can produce all the histological features peculiar to lymphadenoma. (In some cases there is apparently a double infection.) In all cases of doubt resource should be had to the opinion of an experienced morbid anatomist, and, when possible, to an animal experiment. It is stated that antiformin treatment of the tissue after mincing by means of a freezing microtome will result in demonstration of bacilli where other methods have failed. But the writer has had no personal experience with this method.

